# Eleazar Parmly

**Published:** 1875-03

**Authors:** 


### OBITUARY.

Eleazar Parmly, M. D., D. D. S., whose death from pneumonia occurred on the 13th of December, 1874, was a native of Vermont, born in the year 1797, the third of five brothers, four of whom became dentists. Dr. Parmly at the time of his death was the oldest living practitioner of dentistry in this country, and commenced the study of his profession in Montreal, Canada, and afterwards with his brother Dr. Levi Parmly, in New Orleans.

In the year 1820 he proceeded to Europe and practised in London, but returned to this country in 1823, and became one of the most successful practitioners in New York city, where he stood at the head of his profession for more than thirty years.

In the year 1847, Dr. Parmly was elected Provost of the Baltimore College of Dental Surgery, having received from this institution his degree of D. D. S. Dr. Parmly was a very intimate friend of the late President of the College, Prof. Chapin A. Harris, M. D., D. D. S., and also of the first President, Prof. Horace H. Hayden, M. D.

In the year 1861 he gave up practice, although he ever afterwards manifested great interest in the welfare of dentistry.

He was distinguished for his virtues and amiability, and was in every respect a gentleman.

The following resolutions were adopted by the New York Odontological Society :

*Resolved*, That we sincerely mourn the death of our honored friend and professional brother, Eleazer Parmly.

*Resolved*, That we and the public have lost in him one who in past years was the first practitioner of our city, and the chief ornament of our profession, at once an able and distinguished dentist and Christian gentleman ; one who honored and elevated his profession, and did more than any other in his time to make it respected by the community ; one whose character and career is an encouraging example to the young men of the profession ; whose beneficient influence must endure so long as American dentistry is known, and wherever its ministrations are enjoyed.

*Resolved*, That we deeply sympathize with the family of the deceased, and that a copy of these resolutions be presented to them and furnished for publication.

